# Beyond the Anticodon: tRNA Core Modifications and Their Impact on Structure, Translation and Stress Adaptation

**DOI:** 10.3390/genes15030374

**Published:** 2024-03-19

**Authors:** Marcel-Joseph Yared, Agathe Marcelot, Pierre Barraud

**Affiliations:** Expression Génétique Microbienne, Université Paris Cité, CNRS, Institut de Biologie Physico-Chimique, F-75005 Paris, France; marcel.yared@ibpc.fr (M.-J.Y.); agathe.marcelot@ibpc.fr (A.M.)

**Keywords:** transfer RNAs, post-transcriptional modifications, tRNA modifications, tRNA stability, modification circuits, translation, stress response, bacteria, yeast

## Abstract

Transfer RNAs (tRNAs) are heavily decorated with post-transcriptional chemical modifications. Approximately 100 different modifications have been identified in tRNAs, and each tRNA typically contains 5–15 modifications that are incorporated at specific sites along the tRNA sequence. These modifications may be classified into two groups according to their position in the three-dimensional tRNA structure, i.e., modifications in the tRNA core and modifications in the anticodon-loop (ACL) region. Since many modified nucleotides in the tRNA core are involved in the formation of tertiary interactions implicated in tRNA folding, these modifications are key to tRNA stability and resistance to RNA decay pathways. In comparison to the extensively studied ACL modifications, tRNA core modifications have generally received less attention, although they have been shown to play important roles beyond tRNA stability. Here, we review and place in perspective selected data on tRNA core modifications. We present their impact on tRNA structure and stability and report how these changes manifest themselves at the functional level in translation, fitness and stress adaptation.

## 1. Introduction

**Figure 1:**
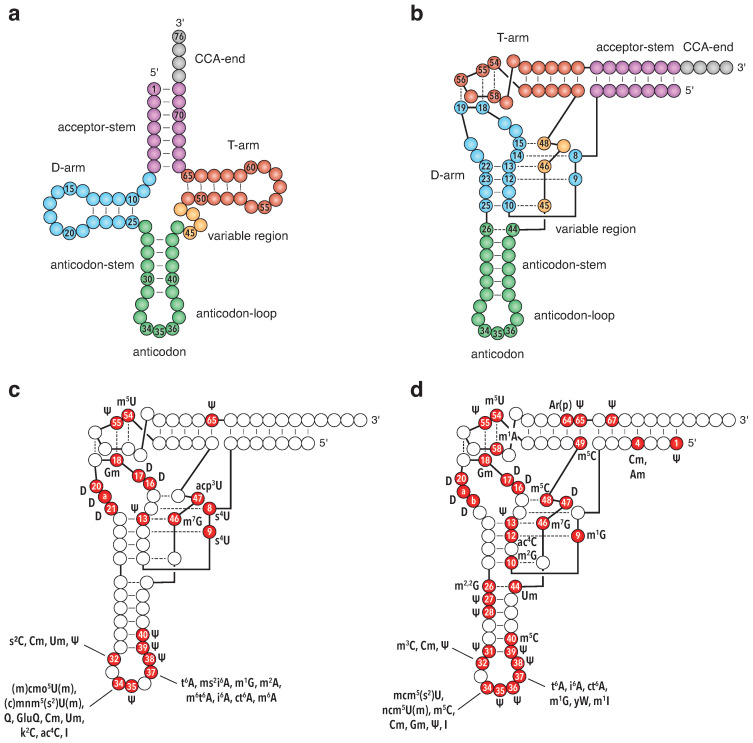
tRNA structure and post-transcriptional modifications: (**a**) Cloverleaf representation of tRNA secondary structure. tRNAs are composed of five main regions, namely the acceptor stem (in purple) with the CCA-end (in grey), the D-arm consisting of the D-loop and D-stem (in blue), the anticodon-arm (in green), the variable region (in yellow), and the T-arm consisting of the T-loop and T-stem (in red). Residue numbers are according to the standard tRNA nomenclature [22]. (**b**) Schematic representation of the L-shaped tertiary structure of tRNA with the same color codes as in (**a**). The acceptor-stem stacks on the T-arm, and the D-arm stacks on the anticodon-arm. The T-loop and D-loop interact and form the tRNA elbow structure. Main tertiary interactions between the T- and D-loops and between the variable region and the D-stem are represented with dashed lines, and the positions of the implicated nucleotides are indicated. (**c**) Locations of modifications in *Escherichia coli* tRNAs. (**d**) Locations of modifications in *Saccharomyces cerevisiae* tRNAs. Data are compiled from the MODOMICS database [14] and references [23,24] for *E. coli* tRNAs and [1,2] for *S. cerevisiae* tRNAs. Modified positions are represented as red circles on the schematic view of the L-shaped tertiary structure of tRNA. Residue numbers and modifications found in *E. coli* and *S. cerevisiae* are given for each modified position.

Transfer RNAs (tRNAs) serve as adaptor molecules during the translation process, where they deliver amino acids to the ribosome and hold a central role in decoding the genetic information contained in messenger RNAs (mRNAs). To fulfill this fundamental function in cellular protein synthesis, tRNAs are produced following a multi-step biogenesis process that leads to the formation of functional tRNAs [1,2,3,4]. Mature tRNAs are generally ∼75–95 nucleotides in length and adopt a cloverleaf secondary structure. This structure defines several regions: the acceptor stem; the D-stem and D-loop (also referred to as the D-arm); the anticodon-stem and anticodon-loop (ACL); the variable region (which usually contains 4 nucleotides but can contain ∼10–25 nucleotides in certain tRNAs, e.g., tRNA^Ser^, tRNA^Leu^ and tRNA^Tyr^); the T-stem and T-loop (i.e., the T-arm); and the CCA-end (Figure 1a) [5,6]. This secondary structure assembly folds into a uniform three-dimensional structure: the L-shaped architecture. This architectural hallmark, in which the T-stem is stacked on the acceptor stem and the D-stem is stacked on the anticodon-stem, is assembled as a result of multiple tertiary interactions between the T- and D-loops, thereby forming the tRNA elbow, and between the variable region and the D-stem (Figure 1b) [7,8,9,10,11].

A striking feature of tRNAs is their universal incorporation of post-transcriptional chemical modifications, with tRNAs being the RNA family that exhibits both the greatest diversity of modifications and the greatest number of modifications per molecule [12,13]. Although ∼100 different modifications have been identified in tRNAs, each tRNA typically contains from 5 to 15 modifications that are incorporated at specific sites along the tRNA sequence [14,15]. Many modified nucleotides are implicated in the formation of the tertiary interactions that lead to the L-shaped structure of tRNAs (see, for instance, the position of modified nucleotides in *E. coli* and *S. cerevisiae*—Figure 1c,d) and are thus key to tRNA stability [16,17] and resistance to RNA decay pathways [18,19,20,21]. Based on their position in the three-dimensional structure, modifications can be divided into two groups, namely modifications in the tRNA core and modifications in the ACL region (Figure 1).

The implication of ACL modifications in the decoding process has been extensively studied, providing many details on the function and dysfunction associated with these modifications (reviewed in [25,26,27,28,29]). The ACL contains complex tRNA modifications that are involved in codon recognition (e.g., at wobble position 34) and reading frame maintenance (e.g., at position 37) [30,31,32,33,34,35,36,37]. Modifications at the wobble position can either restrict or expand the decoding properties of the tRNA, and modifications in the ACL are thus intricately linked to the efficiency and accuracy of translation. Thus, modifications in the ACL can modulate the expression of specific genes that are enriched in codons decoded by tRNAs modified at the wobble position. This notion of modification-tunable expression of codon-biased genes allows for an additional layer of regulation in the gene expression process, which may have general implications for cellular physiology and stress adaptation (reviewed in [38,39,40,41]).

**Figure 2:**
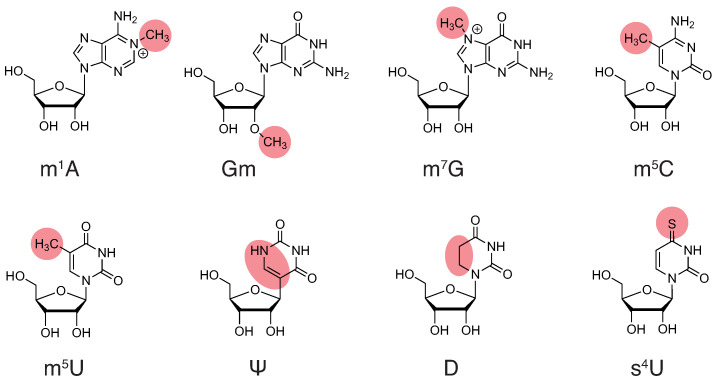
Chemical structure of common modified residues discussed in this review: 1-methyladenosine ($m^{1}$A), 2′-O-methylguanosine (Gm), 7-methylguanosine ($m^{7}$G), 5-methylcytidine ($m^{5}$C), 5-methyluridine ($m^{5}$U), pseudouridine (Ψ), dihydrouridine (D) and 4-thiouridine ($s^{4}$U). Modifications are highlighted in red.

Modifications in the tRNA core, i.e., outside the ACL region, are also implicated in several important cellular functions, such as translation, fitness and stress adaptation. However, the molecular mechanisms involved are generally less well characterized and, therefore, more difficult to comprehend than those involving modifications in the ACL. In addition, in bacteria and yeast, most essential genes, or genes whose deletion leads to marked growth phenotypes, are involved in the introduction of modifications at positions 32, 34 or 37 in the ACL. These positions and their respective modifications have therefore been the focus of many of the studies on tRNA modifications and their associated functions. Overall, modifications of the tRNA core may have been neglected in comparison. In recent years, however, a number of studies have sought to characterize and understand the role of some of these modifications (a few examples are shown in Figure 2). Some tRNA core modifications appear to have roles of their own, with important functions beyond tRNA stability.

The present review aims to bring together and put into perspective selected data on tRNA core modifications. We focus on modifications of cytoplasmic tRNAs in bacteria and yeast. We may take a few detours into higher eukaryotes, but readers interested in these topics are advised to consult more appropriate reviews [42,43,44]. We first present the impact of these modifications on tRNA structure and stability and the hierarchical introduction of other modifications. We then report and discuss how these changes manifest themselves at the functional level and examine their impact on translation, fitness and stress adaptation. Overall, we aim to emphasize the importance of tRNA core modifications, which should not be overlooked in favor of their anticodon-loop counterparts.

## 2. Influence of tRNA Core Modification on tRNA Structure and Stability

### 2.1. Modifications and Their Effect on the Physicochemical Properties of the Nucleotides

The majority of the basic knowledge concerning the impact of core modifications on tRNA structure was mainly discovered towards the end of the last century and has been comprehensively reviewed and discussed elsewhere [16,17,45,46,47,48]. We provide here only a concise overview of these fundamentals, which are at the root of all local and global molecular changes associated with the introduction of modifications.

Pseudouridylation, 2′-O-methylation and 2-thiolation enhance tRNA structural stability through stabilization of the C3′-endo conformation of the ribose moiety and improvement of the base stacking properties of these modified nucleotides [49,50,51,52]. This enhances the thermostability of tRNAs containing these modifications. For instance, Ψ55, Ψ40 and Gm18 individually increase the melting temperature of *E. coli* tRNA^Ser^ [53], and m^5^s^2^U54 increases the melting temperature of thermophile tRNAs [54,55]. In addition, 4-thiolation, such as s^4^U8, also increases tRNA melting temperatures [53], probably by strongly reinforcing the s^4^U8-A14 reverse Hoogsteen base pair (Figure 1c) [56]. Furthermore, as Gm18 and Ψ55 are involved in a tertiary interaction between the D- and T-arms of the tRNAs (Figure 1c,d), they are implicated in stabilizing their global tertiary structure and, more particularly, their elbow region [57]. Pseudouridine exhibits a third stabilizing effect by providing an additional hydrogen-bond donor (H-N1) that may form a water-mediated interaction with the phosphate backbone [52,58,59]. This additional link increases the rigidity of the local tRNA structure and may contribute to the proper tertiary folding of tRNAs [60].

Simple methylations such as m^5^U or m^5^C also exhibit stabilizing properties due to their increased hydrophobicity, increased base polarizability, increased stacking capabilities and reinforcement of m^5^U54-m^1^A58 and G15-m^5^C48 tertiary interactions (Figure 1d) [60,61,62]. This explains why the presence of m^5^U54 increases the melting temperature of tRNAs [53,54]. Other methylations such as m^1^A58 and m^7^G46 introduce a positive charge at the purine aromatic ring of the base, which may stabilize the tRNA structure by interacting with negatively charged phosphates ions of the tRNA backbone or by stabilizing certain tertiary interactions, such as the C13-G22-m^7^G46 base triplet (Figure 1c,d) [47,63]. Other methylations, such as m^1^A9 and $m_{2}^{2}$G26, disrupt the Watson–Crick base-pairing capacity of these nucleotides and are individually essential for the formation of the proper cloverleaf secondary structure and three-dimensional folding of certain tRNAs [64,65,66].

Only the dihydrouridine (D) modification has the capacity to enhance tRNA structural flexibility. Dihydrouridines effectively promote the C2′-endo sugar conformation and prevent proper stacking interaction with neighboring bases, thereby allowing for greater conformational flexibility and dynamic motion in the tRNA D-loop (reviewed in [67]).

### 2.2. Modifications in the tRNA Core and Their Impact on tRNA Structure and Dynamics

First, it is important to note that tRNA modifications can have little to no effect on some tRNAs while dramatically affecting others. For instance, in the yeast elongator ${\mathrm{tRNA}}^{\mathrm{Phe}}$, the introduction of m^1^A58 has no detectable impact on the structure of the tRNA as observed with NMR [68,69]. On the contrary, the introduction of m^1^A58 on in vitro transcribed unmodified yeast ${\mathrm{tRNA}}_{i}^{\mathrm{Met}}$ has a major effect on the tertiary folding of this tRNA and more particularly on the proper assembly of its elbow region [70]. NMR studies showed that unmodified ${\mathrm{tRNA}}_{i}^{\mathrm{Met}}$ adopts different heterogeneously folded conformations while m^1^A58-${\mathrm{tRNA}}_{i}^{\mathrm{Met}}$ adopts one major homogenously folded conformation. This important structural role of m^1^A58 in ${\mathrm{tRNA}}_{i}^{\mathrm{Met}}$, which has a structural impact reminiscent of that of m^1^A9 and $m_{2}^{2}$G26, is likely related to the unique tRNA substructure in the elbow region of eukaryotic initiator tRNAs [71]. It should be emphasized that the tRNA-dependent nature of the impact of modifications is perfectly in line with the idea that certain modifications do not necessarily have the same beneficial effect and are not equally useful for all tRNAs (reviewed in [72]).

Although dihydrouridines are considered to bring flexibility to tRNAs, their effects can be ambivalent. Dihydrouridines indeed allow greater conformational flexibility and dynamic motion in the tRNA D-loop [73] but also simultaneously constrain the D-stem to adopt a stable conformation, as seen in *S. pombe* ${\mathrm{tRNA}}_{i}^{\mathrm{Met}}$ [74]. The stabilization effect of dihydrouridines is also observed in *E. coli* tRNA^Ser^ where the absence of D20 decreases the melting temperature of this tRNA [53]. Lastly, it has been suggested that the interplay between the flexibility of D20 and the rigidity of Ψ55 is needed to stabilize the elbow region of *E. coli* ${\mathrm{tRNA}}_{f}^{\mathrm{Met}}$ [75]. Another example affecting the melting temperature of tRNAs concerns the ${\mathrm{acp}}^{3}$U47 core modification, which increases the thermal stability of *E. coli* ${\mathrm{tRNA}}^{\mathrm{Met}}$ by 3 °C [76]. The exact mechanism of how ${\mathrm{acp}}^{3}$U47 stabilizes the tertiary structure of tRNAs is not well understood. However, it has been suggested that it could be due to its inability to form Watson–Crick base pairing, thus ensuring the correct folding of tRNAs [76].

Although chemical modifications of nucleotides often increase the overall thermal stability of tRNAs, this may be achieved by an increase or a decrease in the local conformational dynamics in solution [45]. For instance, modified *E. coli* ${\mathrm{tRNA}}_{f}^{\mathrm{Met}}$ has a higher overall structural and thermal stability, compared to its unmodified version, but presents an increase in local conformational fluctuations [75]. This behavior is attributed to entropic effects in which increased local conformational dynamics lead to a stabilization of the tertiary structure of the tRNA [75], in line with previous molecular dynamics simulations [77,78]. Interestingly, the authors observed that tRNA core modifications generate a remote effect of stabilization throughout the entire tRNA [75], which correlates well with an earlier report stating that modifications alter the intrinsic correlated motions within the tRNAs [52]. Effects of tRNA core modifications on the conformational dynamics of tRNAs are likely tRNA-dependent, and fully modified human mt-${\mathrm{tRNA}}^{\mathrm{Leu}}$, for instance, displays lesser folding dynamics than the unmodified transcript and exhibits a more constrained native structure that does not allow intermediate conformations [79].

### 2.3. Influence of tRNA Core Modification Enzymes on tRNA Structure: More Than Just Their Catalytic Activity

Modification enzymes have been reported to act as chaperones stabilizing and refolding their substrates independently of their enzymatic activity, thereby giving misfolded tRNAs a second chance to refold into native conformations (reviewed in [80]). Two tRNA modification enzymes acting as chaperones have been identified and characterized in *E. coli*, namely TruB introducing Ψ55 and TrmA introducing m^5^U54 [81,82]. The chaperone activity of both of these enzymes comes from their ability to partially unfold and open the tRNA elbow region by disrupting tertiary interactions. It is thus likely that other modification enzymes possessing the capacity to unfold the tRNA elbow region would also act as tRNA-specific chaperones independently of their catalytic activity [83,84]. For instance, eukaryotic fission yeast Trm1 has been recently reported to have a chaperone-like activity similar to prokaryotic TruB and TrmA [85]. Trm1 enhances tRNA functionality in vivo, even in the absence of catalytic activity, and promotes RNA strand annealing and dissociation, as previously reported for other RNA chaperones [85,86].

It is worth mentioning that the RNA chaperone La, implicated in tRNA 3′-end protection and pre-tRNA folding, shares some functional redundancy with tRNA modification enzymes displaying chaperone activity, such as Trm1 and Pus4 [85,87,88]. The La chaperone does not discriminate between misfolded and properly folded tRNAs (as was also observed for the modification enzyme TruB [81,89]), which creates potential challenges for tRNA chaperones to bind and refold all defective substrates.

### 2.4. tRNA Core Modifications and Their Impact on tRNA Degradation and Cellular Stability

As described above, the absence of certain modifications may alter the folding and tertiary structure of tRNAs. The recognition and elimination of these hypomodified tRNAs with compromised tertiary structures are carried out by tRNA quality control pathways. They prevent hypomodified tRNAs from massively entering the translation process. The characterization of the pathways targeting the degradation of tRNAs lacking specific modifications is much more advanced in eukaryotes, but similar mechanisms seem to also exist in bacteria (see below).

In eukaryotes, two main pathways for tRNA degradation have been identified: the nuclear surveillance pathway, which primarily degrades defective tRNA precursors (pre-tRNAs) in the nucleus, and the Rapid tRNA Decay (RTD) pathway, which degrades hypomodified mature tRNAs in the cytoplasm (reviewed in [1]). Nuclear surveillance and RTD degradation pathways have been extensively studied in the yeasts *S. cerevisiae* and *S. pombe*, where several hypomodified tRNAs have been correlated with phenotypes of thermosensitivity and growth defects (see Table 1 for an overview of the tRNAs targeted by degradation pathways and the tRNA core modifications involved).

Early reports showed that yeast pre-${\mathrm{tRNA}}_{i}^{\mathrm{Met}}$ lacking m^1^A58 is degraded in the nucleus by the nuclear surveillance pathway [19,90]. However, a more recent study revealed that a fraction of this hypomodified initiator tRNA continues the maturation process and is exported to the cytoplasm, where it is given a second chance to be degraded by the RTD pathway (Table 1) [91]. The latter study also showed that *S. pombe* ${\mathrm{tRNA}}_{i}^{\mathrm{Met}}$ lacking m^1^A58 is only degraded by the RTD pathway and not by the nuclear surveillance pathway (Table 1) [91]. As mentioned earlier, m^1^A58 has a drastic impact on the tertiary structure of yeast ${\mathrm{tRNA}}_{i}^{\mathrm{Met}}$, so that the tRNA elbow structure is only properly assembled when this modification is present [70]. This provides a structural explanation of why hypomodified ${\mathrm{tRNA}}_{i}^{\mathrm{Met}}$ lacking m^1^A58 is targeted by RNA decay pathways. This is also in line with a genetic screen, which reported that degradation by the RTD pathway in yeast primarily depends on the structural integrity and stability of the acceptor-stem and T-arm region of tRNAs [92].

Recently, a study reported a new tRNA quality control mechanism in *S. cerevisiae*, in which mature ${\mathrm{tRNA}}^{\mathrm{Tyr}}$ lacking m^1^G9 is eliminated by a Met22-dependent but RTD- and nuclear surveillance-independent degradation pathway (Table 1) [93]. Further investigation is definitely needed to identify the nucleases responsible for this degradation and the molecular mechanisms of this intriguing tRNA quality control pathway. Interestingly, m^1^G9 prevents the degradation of ${\mathrm{tRNA}}^{\mathrm{Tyr}}$, whereas its absence in ${\mathrm{tRNA}}^{\mathrm{Gly}}$ has no effect on its cellular stability [93], adding to the list of known examples of tRNA core modifications that have different effects depending on the tRNA species and are only required for the cellular stability of a particular tRNA [72].

**Figure 3:**
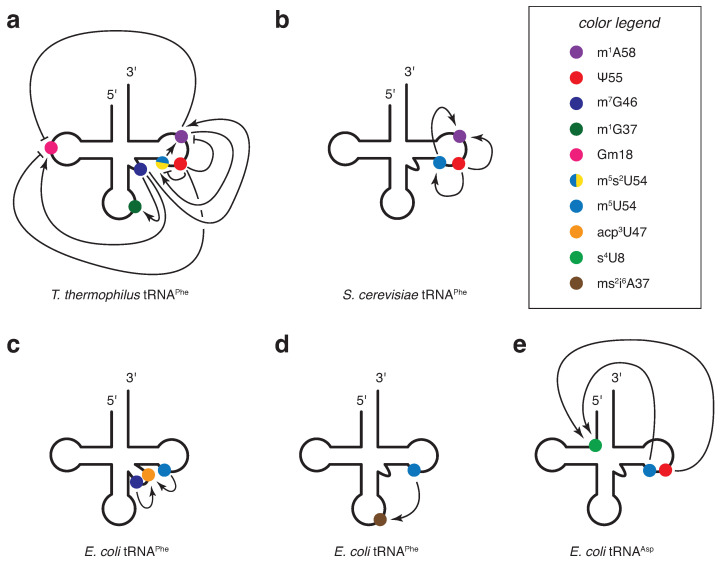
Modification circuits involving tRNA core modifications: (**a**) modification circuits identified in *T. thermophilus* ${\mathrm{tRNA}}^{\mathrm{Phe}}$ in response to temperature changes represented on a cloverleaf scheme [94,95,107]; (**b**) T-arm modification circuits identified in yeast ${\mathrm{tRNA}}^{\mathrm{Phe}}$ [69,70]; (**c**,**d**) modification circuits identified in *E. coli* ${\mathrm{tRNA}}^{\mathrm{Phe}}$ [109,110,111]; and (**e**) modification circuits identified in *E. coli* ${\mathrm{tRNA}}^{\mathrm{Asp}}$ [111]. Modifications are represented by colored circles (see the legend in the box for the corresponding colors). Arrows indicate stimulatory effects between modifications, and blunted lines indicate inhibitory effects between modifications.

In bacteria, tRNA surveillance and quality control systems monitoring hypomodified and/or thermodynamically destabilized tRNAs have not been studied extensively. These aspects were initially studied in thermophilic bacteria, where tRNA thermal and structural stability are required for optimal cellular thermotolerance and survival at elevated growth temperatures [94]. The tRNA core modifications and their dynamic regulation represent an important player in the structural and thermal adaptation of tRNAs (Figure 3a), and their absence may lead to the targeting of the hypomodified tRNA to quality control pathways. For instance, the lack of m^7^G46 in *T. thermophilus* tRNAs leads to the degradation of ${\mathrm{tRNA}}^{\mathrm{Phe}}$ and ${\mathrm{tRNA}}^{\mathrm{Ile}}$ by a yet unidentified pathway (Table 1) [95]. Another important report revealed a bacterial tRNA quality control system in *Vibrio cholerae* where hypomodified tRNAs lacking s^4^U8 are rapidly eliminated by the RNA degradosome (Table 1) [20]. The similarities observed with the well-characterized mechanisms present in eukaryotes definitely call for further studies in other bacterial species, in order to refine our understanding of the role of tRNA core modifications in the cellular stability of tRNAs in bacteria.

**Table 1:** List of eukaryotic and bacterial hypomodified tRNAs that are degraded by tRNA quality control pathways.

Organism	tRNA	Lacking Modifications	Conditions	Degradation Pathway	References
*S. cerevisiae*	pre-${\mathrm{tRNA}}_{i}^{\mathrm{Met}}$	$m^{1}$A58	36 °C	Nuclear surveillance	[19,90]
*S. cerevisiae*	${\mathrm{tRNA}}_{i}^{\mathrm{Met}}$	$m^{1}$A58	27 °C, 34 °C	RTD	[91]
*S. pombe*	${\mathrm{tRNA}}_{i}^{\mathrm{Met}}$	$m^{1}$A58	30 °C, 38.5 °C	RTD	[91]
*S. cerevisiae*	${\mathrm{tRNA}}^{\mathrm{Tyr}}$	$m^{1}$G9	30 °C, late log phase, and w/ or w/o 5FU	Met22-dependent but Xrn1, Rat1, Dxo1, Trf4 and Rrp6-independent degradation	[93]
*S. cerevisiae*	${\mathrm{tRNA}}^{\mathrm{Val}(\mathrm{AAC})}$	$m^{7}$G46 and ($m^{5}$C or Ψ13 or D47) *	37 °C	RTD	[18,96]
*S. cerevisiae*	${\mathrm{tRNA}}^{\mathrm{Ser}(\mathrm{CGA},\mathrm{UGA})}$	Um44 and ${\mathrm{ac}}^{4}$C12 *	36.5 °C	RTD	[96,97]
*S. cerevisiae*	${\mathrm{tRNA}}^{\mathrm{Ser}(\mathrm{CGA},\mathrm{UGA})}$	$m_{2}^{2}$G26 and $m^{5}$C *	37 °C	RTD	[98]
*S. cerevisiae*	${\mathrm{tRNA}}^{\mathrm{Val}(\mathrm{AAC})}$	$m^{7}$G46	37 °C after thiolutin treatment	RTD	[98]
*S. cerevisiae*	${\mathrm{tRNA}}^{\mathrm{Ser}(\mathrm{CGA},\mathrm{UGA})}$	${\mathrm{ac}}^{4}$C12	37 °C after thiolutin treatment	RTD	[98]
*S. cerevisiae*	${\mathrm{tRNA}}^{\mathrm{Ser}(\mathrm{CGA},\mathrm{UGA})}$	$m_{2}^{2}$G26	37 °C	RTD	[98]
*S. pombe*	${\mathrm{tRNA}}^{\mathrm{Tyr}(\mathrm{GUA})}$ and ${\mathrm{tRNA}}^{\mathrm{Pro}(\mathrm{AGG})}$	$m^{7}$G46	36.5 °C, 37.5 °C, 38.5 °C	RTD	[99]
*T. thermophilus*	${\mathrm{tRNA}}^{\mathrm{Phe}}$ and ${\mathrm{tRNA}}^{\mathrm{Ile}}$	$m^{7}$G46	70–80 °C	-	[95]
*V. cholerae*	${\mathrm{tRNA}}^{\mathrm{Tyr}}$, ${\mathrm{tRNA}}_{1}^{\mathrm{Ser}}$, ${\mathrm{tRNA}}_{1}^{\mathrm{Cys}}$, ${\mathrm{tRNA}}_{2}^{\mathrm{Cys}}$ and others	$s^{4}$U8	stationary phase at 37 °C	RNA degradosome	[20]
*V. cholerae*	${\mathrm{tRNA}}_{1A}^{\mathrm{Gln}}$	$s^{4}$U8	log phase at 37 °C	RNA degradosome	[20]
*V. cholerae*	${\mathrm{tRNA}}^{\mathrm{Tyr}}$	$s^{4}$U8 and (Ψ55 or $m^{5}$U54) *	log phase at 37 °C and w/o arabinose	RNA degradosome	[20]

* Both listed modifications should be absent for the tRNA to be degraded.

### 2.5. Interdependence Involving tRNA Core Modifications

Although some modifications are incorporated into tRNAs independently, modification circuits have been identified in which the prior presence of one modification influences the occurrence and incorporation of subsequent modifications [3,100,101,102]. Most of the well-characterized modification circuits concern modifications in the ACL region. Few examples involving tRNA core modifications have been reported, both in bacteria and yeast (Figure 3). The mechanisms underlying the altered efficiency of tRNA modification incorporation by initial modifications are not yet well understood, but it is reasonable to propose that alterations in the local structure and/or dynamics of the tRNA would affect the catalytic efficiency of the subsequent enzymes. These alterations could occur at all steps of the catalytic cycle of the modification enzymes, from substrate binding to substrate release.

The first tRNA core modification circuits identified were those found in *T. thermophilus* ${\mathrm{tRNA}}^{\mathrm{Phe}}$ (Figure 3a). They regulate modification levels overall in response to temperature changes (reviewed in [94]). Briefly, at elevated temperatures, the prior presence of $m^{7}$G46 promotes the introduction of Gm18 and m^1^A58, which subsequently promotes the incorporation of m^5^s^2^U54 [95,103], with m^1^A58 and m^5^s^2^U54 being essential for the growth of several thermophiles at high temperatures [104,105,106]. In addition, m^7^G46 promotes the incorporation of m^1^G37 in the ACL [95]. However, at low temperatures, the prior presence of Ψ55 inhibits the introduction of Gm18, m^1^A58 and m^5^s^2^U54, maintaining sufficient tRNA structural flexibility to survive at lower temperatures [107]. In addition, at low temperatures, m^5^U54 stimulates the introduction of m^1^A58 [108].

In the yeast *S. cerevisiae*, the time-resolved monitoring with NMR spectroscopy of the maturation of a ${\mathrm{tRNA}}^{\mathrm{Phe}}$ transcript in yeast cell extracts revealed the existence of dependencies between tRNA core modifications [69]. In particular, a strong modification circuit is present in the T-arm, in which the prior presence of Ψ55 activates the formation of m^5^U54 by Trm2 and m^1^A58 by Trm6/Trm61, and m^5^U54 promotes the formation of m^1^A58 (Figure 3b). These cross-talks were shown to be also present in other yeast tRNAs, although they were not specifically identified [69]. A recent study conducted nanopore sequencing of all yeast tRNAs in specific deletion strains [112]. This study confirmed the modification circuit in the T-arm of ${\mathrm{tRNA}}^{\mathrm{Phe}}$ and further showed that it is also present in at least 15 other tRNA species [112]. Further biochemical studies on this Ψ55 → m^5^U54 → m^1^A58 modification circuit, have characterized and quantified the direct effect of the initial modifications on the enzymatic activity of the subsequent ones [70]. The Ψ55 and m^5^U54 modifications have a strong cumulative effect on the introduction of m^1^A58, together accelerating this process by a factor of ∼15 (discussed in [113]).

Recently, several reports have shed light on modification cross-talks among tRNA core modifications in *E. coli*. They reported that the introduction of ${\mathrm{acp}}^{3}$U47 is globally enhanced by the prior presence of m^7^G46 [109] and also stimulated by m^5^U54 in *E. coli* ${\mathrm{tRNA}}^{\mathrm{Arg}}$, ${\mathrm{tRNA}}^{\mathrm{Ile}(\mathrm{GAU})}$, ${\mathrm{tRNA}}^{\mathrm{Lys}}$, ${\mathrm{tRNA}}^{\mathrm{Phe}}$ and ${\mathrm{tRNA}}^{\mathrm{Val}}$ [110,111] (Figure 3c). Ψ55 seems to also participate in this network, in which it may either slightly stimulate or inhibit the introduction of ${\mathrm{acp}}^{3}$U47 in *E. coli* ${\mathrm{tRNA}}^{\mathrm{Phe}}$ and ${\mathrm{tRNA}}^{\mathrm{Lys}}$, respectively [111]. In addition, the formation of ms^2^$i^{6}$A37 in the ACL of *E. coli* ${\mathrm{tRNA}}^{\mathrm{Phe}}$ is activated by m^5^U54 [110,111] (Figure 3d), and the introduction of s^4^U8 is enhanced in the presence of Ψ55 and m^5^U54 in *E. coli* ${\mathrm{tRNA}}^{\mathrm{Asp}}$ [111] (Figure 3e). These interdependencies in *E. coli* tRNA core modifications were identified using a combination of nucleotide quantification and sequencing techniques, such as HPLC, liquid-chromatography coupled to tandem mass spectrometry (LC-MS/MS), multiplex small RNA-sequencing (MSR-seq) and primer extension analysis. Finally, an order for the introduction of modifications into *E. coli* ${\mathrm{tRNA}}^{\mathrm{Phe}}$ was suggested where Ψ55 and m^5^U54 are introduced in the earliest stages of maturation, followed by m^7^G46 and then ${\mathrm{acp}}^{3}$U47, with s^4^U8 being introduced after Ψ55 and m^5^U54 [114].

Apart from the few examples described above, the characterization of these interdependencies is still relatively limited, but the latest technological developments, in particular in nanopore sequencing technology, are expected to accelerate the discovery of modification circuits involving tRNA core modifications [115].

## 3. tRNA Core Modifications and Their Impact on Translation and Stress Adaptation

### 3.1. tRNA Modification Levels Depend on Growth Conditions

Although tRNA modifications may appear to be constitutively introduced as static decoration on every tRNA of the cell, the level and/or nature of certain modifications in a tRNA population may change in response to environmental factors such as growth phase, temperature, cellular stresses or nutrient availability. These variations consist of the appearance, disappearance or change in the nature of the modification at the individual tRNA molecule level. Collectively, these variations have an impact on the level of modifications in tRNA populations [3].

During the exponential growth phase, most microorganisms double their content on an hourly basis. In *E. coli*, the tRNA pool does not increase homogeneously during exponential growth. Instead, rare tRNAs tend to decrease in concentration while the concentration of other tRNAs remains apparently constant [116,117,118]. In *S. cerevisiae*, a similar phenomenon exists where tRNA proportions vary significantly upon stress, which may be part of stress response mechanisms [119]. These changes in tRNA proportions are linked to changes in tRNA modification status. Some abundant tRNAs are hypomodified during the exponential phase and complete their maturation during the stationary phase [116,120]. In *E. coli*, abundant tRNAs tend to be less fully thiolated than tRNAs with rare codons [116]. In *B. subtilis*, more complete modifications are found in tRNAs from stationary cells in comparison with tRNAs of exponentially growing cells [120].

In addition to the growth phase, growth conditions such as stress exposure, have been shown to affect tRNA modification levels in microorganisms. A pioneering study used HPLC-coupled mass spectrometry to quantitatively analyze modifications in tRNAs. The study showed that exposure to toxic substances such as methylmethane sulfonate (MMS), hydrogen peroxide ($H_{2}$$O_{2}$), sodium arsenite (NaAs$O_{2}$) and sodium hypochlorite (NaOCl) in *S. cerevisiae* affects the level of several modifications, many of which being tRNA core modifications [121]. Upon treatment with $H_{2}$$O_{2}$, levels of m^5^C and $m_{2}^{2}$G were increased, whereas levels of m^5^U, m^2^G and m^1^A were slightly decreased [121]. Various toxicants induced similar yet distinct changes in tRNA modifications, indicating that dynamic changes in tRNA modifications in response to cellular stresses are widespread and stress specific [121,122]. Although the roles of these changes are still mostly unknown, some changes occurring at the wobble position have been interpreted in terms of modification-tunable expression of codon-biased genes (reviewed in [38,39,40,41]). The possible role of the changes affecting tRNA core modifications upon stress exposure remains much less well characterized in comparison.

Several other studies have reported alterations in the levels of tRNA core modifications upon exposure to various stresses or growth conditions. This includes, for instance, the Gm18 modification that is increased in *E. coli* upon mild antibiotic stress [123]. In *S. cerevisiae*, additional m^5^C modifications are specifically introduced on ${\mathrm{tRNA}}^{\mathrm{His}}$ at positions 48 and 50 in response to conditions characterized by global cell growth arrest [124]. In *T. thermophilus*, changes in the growth temperature were shown to affect levels of tRNA core modifications (see text above and references [94,106]). In *E. coli*, heat stress and/or heat shock were also found to either increase or decrease the levels of several tRNA core modifications, e.g., s^4^U8/9, Gm18, m^7^G46 and ${\mathrm{acp}}^{3}$U47 [125]. These examples show that it is important to bear in mind that tRNA core modifications also undergo changes in response to stressful conditions and that this phenomenon is not limited to the modifications found at the wobble position.

### 3.2. tRNA Core Modifications and Their Impact on Translation and Fitness

The binding of tRNAs to the ribosome and their accommodation into the A-site constitute the first steps of translation elongation. The reaction only becomes irreversible when fitting tRNAs enter the A-site [126], making accommodation a limiting step for translation elongation [126,127]. Inside the A-site, tRNAs interact with the mRNA codon as well as the small ribosomal subunit via their anticodon-arm, leading to the well-characterized proofreading mechanism, and with the large ribosomal subunit mainly via their T-arm and elbow region [11,128,129,130]. After the accommodation of the incoming tRNA into the A-site and the catalysis of the peptide transfer from the P- to the A-site tRNA, the peptidyl A-site tRNA is translocated to the P-site. During the translocation process, tRNAs also interact with the ribosome through their T-arm and elbow regions [11,129,131]. Modifications in the tRNA core that affect the structure and dynamics of tRNAs may therefore affect the interaction with the ribosome and modulate the efficiency of translation elongation by affecting accommodation and/or translocation.

Several recent studies have shed new light on the mechanisms by which modifications in the tRNA core impact fitness and translation [110,111,132]. Effects of certain tRNA core modifications on fitness have been known for many years. For instance, in *E. coli*, the *trmA* and *truB* genes, responsible for the introduction of m^5^U54 and Ψ55 in the T-loop, are non-essential under most growth conditions, but subtle growth phenotypes have been reported for the *trmA*Δ and *truB*Δ strains [57,133]. In addition, these genes are important for bacterial fitness in co-culture experiments [134,135]. The fitness phenotypes in *E. coli* are attributed to the chaperone activity of TruB [81], while both the chaperone and modification activities of TrmA are implicated [82]. The effect of m^5^U54 on translation was initially investigated in vitro with *E. coli* tRNAs, which pointed towards a role for m^5^U54 in both translation speed and fidelity [136]. The binding to the A-site of a ${\mathrm{tRNA}}^{\mathrm{Lys}(\mathrm{UUU})}$ lacking m^5^U54 was reduced, and the resulting translation speed increased by ∼10-fold, while translation with ${\mathrm{tRNA}}^{\mathrm{Phe}(\mathrm{GAA})}$ displayed about a ∼10-fold loss of fidelity [136]. In yeast, there is a slight global increase in the translation efficiency in *trm2*Δ strains lacking m^5^U54 [137]. It is also worth mentioning that in humans, the m^5^U54 modification appears to be involved in translation fidelity [138].

In a recent report that conducted in vitro kinetic studies of translation with *E. coli* tRNAs prepared from *trmA*Δ cells, the m^5^U54 modification was shown to play a role in the translocation process [110]. The use of hygromycin B—an antibiotic known to block the translocation of ribosomes—led to the proposal that m^5^U54 affects the translocation step and could reduce the translation speed by restraining the translocation of tRNAs [110]. In another report that determined codon-specific changes in translation efficiency in the *trmA*Δ and *truB*Δ single- and double-knockout strains, a set of specific codons affecting the expression of a reporter gene was identified [111]. For instance, in the *truB*Δ strain, a decrease in translation is detected when some arginine codons, i.e., CGU, CGG and AGG, are placed consecutively. Similarly, in the *trmA*Δ strain, translation is decreased at consecutive cysteine TGT codons but increased at consecutive tyrosine TAT codons [111]. Although the overall translation is unaffected, the codon-specific impact related to the loss of m^5^U54 and/or Ψ55 in the *trmA*Δ and *truB*Δ strains leads to changes in the abundance of specific proteins, many of which are implicated in metabolism and gene expression [111]. This decreased synthesis of proteins enriched in the identified codons might explain the fitness phenotype of the *trmA*Δ and *truB*Δ strains. In these two studies focused on the impact of tRNA core modifications on translation [110,111], the reported changes at the level of the translocation step, or related to codon-specific changes in the translation efficiency, could be attributed to subtle differences in the local dynamics of the tRNA elbow region that could help tune the rates of different translation steps, e.g., accommodation and translocation, to ensure a good balance between the speed and fidelity of translation [139].

These observations are perfectly in line with another recent report that investigated in a more general and broader context the effect of tRNA core modifications on translation in *S. cerevisiae* [132]. This report is based on the initial hypothesis that tRNA core modifications may have an impact on the intrinsic decoding efficiency of a tRNA by altering its structure and/or flexibility, which could potentially influence the dynamics of its interaction with the ribosome during accommodation and/or translocation. Mistranslation reporters have been used to directly measure amino acid incorporation errors in vivo, to determine whether the loss of specific tRNA core modifications may affect translational misreading errors [132]. The loss of individual modifications globally led to an increase in the misreading frequency. Each modification affected the misreading error of the selected error-prone codons in an intricate manner, and certain combinations did not give rise to significant changes. Strikingly, however, the absence of almost any of the core modifications, altered the accuracy of the tested tRNAs, i.e., ${\mathrm{tRNA}}^{\mathrm{Lys}}$ and ${\mathrm{tRNA}}^{\mathrm{Glu}}$, on at least one of the error-prone codons tested [132].

These recent findings on the impact of tRNA core modifications on the fine tuning of the translation process [110,111,132], which appear to be modification-, tRNA- and codon-specific, support the idea that the structure of the tRNA core, including its modifications, have coevolved with the anticodon to tune tRNAs with strong or weak anticodons to achieve a consistent efficiency of decoding [140].

### 3.3. tRNA Core Modifications and Their Relation with Stress Adaptation Mechanisms

#### 3.3.1. s^4^U8 Is Implicated in the Response to UV Stress

The $s^{4}$U8 modification is omnipresent in prokaryotic tRNAs [14] and has been reported early on to carry a protective role against UV radiation (Table 2) (reviewed in [141]). This modification is sensitive to UVs [142], and when cytosine is present at position 13, an intramolecular $s^{4}$U8:C13 cyclobutane pyrimidine dimer is formed between the aromatic bases that are stacked on each other in tRNA 3D structures [142]. This UV-triggered cross-linking is a widespread phenomenon, since in *E. coli* for instance, about two-thirds of the tRNAs harbor the $s^{4}$U8 modification, with half of them also carrying cytosine at position 13. Thus, UV radiation likely targets about one-third of the *E. coli* tRNAs, including the entire pool of several isoacceptor tRNAs. Some of these photo-crosslinked tRNAs exhibit a reduced ability to be charged by their aminoacyl-tRNA synthetase [143,144]. The accumulation of uncharged tRNAs—which may be directed to the A-site of the ribosome—causes protein synthesis to stall and leads to growth delay in *E. coli* and *Salmonella typhimurium* [145,146,147]. In addition, ribosome stalling gives rise to the production of ppGpp, which triggers the stringent response and a massive decrease in stable RNA production. This seemingly allows for a faster recovery of the UV-exposed cells upon antibiotic treatment [148]. Interestingly, *E. coli* strains selected for their abnormal sensitivity to UVs, were found to carry mutations in the gene responsible for $s^{4}$U8 formation [149,150] and were unable to produce ppGpp after irradiation [147]. These mechanisms—initiated by a photo-cross-linking reaction at the $s^{4}$U8 modification site—eventually ensure better survival after UV stress [147,150].

#### 3.3.2. Gm18 Regulates the Immune-Stimulatory Effect of tRNAs

The Gm18 modification is present in both bacteria and eukaryotes [14]. In *E. coli*, this modification was recently shown to be involved in a stress adaptation mechanism to antibiotic treatment in relation to the sensing of bacteria by the immune system of the human host (Table 2) [123]. Bacterial tRNAs containing Gm18 exhibit a reduced immunostimulatory effect in humans [151]. Whereas unmodified tRNAs trigger the activation of the human Toll-like receptor 7 (TRL7) [152], Gm18-modified tRNAs act as TRL7 antagonists and inhibit the immune response [153,154]. In *E. coli*, the gene encoding the G18 methyltransferase, i.e., *trmH*, is part of an operon activated during the stringent response, and levels of Gm18 are thus expected to increase in certain stress conditions, for instance antibiotic treatment [155]. An increase in the Gm18 level in some tRNAs was indeed observed upon mild antibiotic treatment in *E. coli* [123]. In addition, and in line with previous reports, the stress-induced increase in tRNA Gm18 levels led to a reduced immune-stimulatory effect of the bacterial tRNAs [123]. Thus, Gm18 is a tRNA core modification that responds to stress with implications outside protein translation and appears as a key modification to escape the immune system of the host.

#### 3.3.3. m^7^G46 Is Implicated in the Response to Oxidative Stress

The $m^{7}$G46 modification is present in both bacteria and eukaryotes [14]. The genes responsible for introducing $m^{7}$G46 in tRNAs, i.e., *trmB* in bacteria and *trm8/trm82* in yeast, are not essential for cell viability in optimal growth conditions [95,156,157]. However, they are important in certain growth conditions or together with the deletion of other tRNA modification genes (Table 1 and Table 2) [18,95,96,98]. In *Pseudomonas aeruginosa*, the $m^{7}$G46 modification has been shown to be involved in an adaptation mechanism to $H_{2}$$O_{2}$ oxidative stress (Table 2) [158]. In a *trmB*Δ strain, translation of phenylalanine and aspartate codons by ${\mathrm{tRNA}}^{\mathrm{Phe}(\mathrm{GAA})}$ and ${\mathrm{tRNA}}^{\mathrm{Asp}(\mathrm{GUC})}$ are less efficient, which has a negative effect on the translation of mRNAs enriched in Phe and Asp. It is worth mentioning that, in humans, the $m^{7}$G46 modification seems important as well for the efficient translation of certain codons [159]. In *P. aeruginosa*, the Phe and Asp codons are particularly enriched and grouped in clusters in the mRNAs of the catalases KatA and KatB, two reductases involved in $H_{2}$$O_{2}$ detoxification. Interestingly, the transcription of *trmB* increases following $H_{2}$$O_{2}$ treatment, which results in an increase in the $m^{7}$G46 level in tRNAs and, subsequently, in an increase in KatA and KatB production [158]. This complete regulatory loop is one of the best characterized examples of a tRNA core modification involved in stress response, although some of the underlying mechanisms are still not fully understood. As expected, the *trmB*Δ mutant strain presents an $H_{2}$$O_{2}$-sensitive phenotype, and the survival rate of *trmB*Δ cells exposed to 20 mM $H_{2}$$O_{2}$ was found to be only 10% of that of the wild-type strain [158]. Interestingly, similar $H_{2}$$O_{2}$-sensitive phenotypes have been reported for *trmB* or *trm8* mutants in *E. coli* [160], in *Acinetobacter baumannii* [161] and in the fungus *Colletotrichum lagenarium* [162], demonstrating the widespread implication of the $m^{7}$G46 tRNA core modification in the response to oxidative stress.

**Table 2:** List of tRNA core modifications linked to stress.

Organism	Modification	Type of Stress	References
*S. cerevisiae*	Am4	MMS, NaOCl	[121]
*E. coli*, *S. typhimurium*	$s^{4}$U8	UV	[147,149,150]
*E. coli*	$s^{4}$U8/$s^{4}$U9	heat	[125]
*S. cerevisiae*	$m^{2}$G10	$H_{2}$$O_{2}$, NaOCl, NaAs$O_{2}$	[121]
*T. thermophilus*, *E. coli*	Gm18	cold/heat	[95,107,125,163]
*E. coli*, *V. cholerae*	Gm18	antibiotics	[123,155,164]
*S. cerevisiae*	Gm18	NaOCl	[121]
*S. cerevisiae*	$m_{2}^{2}$G26	$H_{2}$$O_{2}$, MMS, NaAs$O_{2}$	[121,122]
*S. cerevisiae*	Um44	$H_{2}$$O_{2}$, NaAs$O_{2}$, NaOCl	[121,165]
*C. lagenarium*, *P. aeruginosa*, *A. baumannii*, *E. coli*	$m^{7}$G46	$H_{2}$ $O_{2}$	[158,160,161,162]
*T. thermophilus*, *E. coli*	$m^{7}$G46	heat	[95,125]
*V. cholerae*	$m^{7}$G46	antibiotics	[164]
*S. cerevisiae*	$m^{7}$G46	MMS, NaAs$O_{2}$	[121,165]
*E. coli*	${\mathrm{acp}}^{3}$U47	heat	[125]
*S. cerevisiae*	$m^{5}$C48/$m^{5}$C50	amino acid, glucose and uracil starvation	[124]
*T. thermophilus*	$m^{5}$U54	cold	[107,108]
*P. furiosus*, *T. thermophilus*	$m^{5}$$s^{2}$U54	heat	[95,104,107,166]
*T. thermophilus*	Ψ55	cold/heat	[107]
*V. cholerae*	Ψ55	antibiotics	[164]
*T. thermophilus*	$m^{1}$A58	heat	[95,105,107]
*S. cerevisiae*	$m^{1}$A58	$H_{2}$ $O_{2}$	[121]
*V. cholerae*, *E. coli*	D	antibiotics	[164]
*S. cerevisiae*	$m^{5}$C	$H_{2}$$O_{2}$, MMS, NaOCl, NaAs$O_{2}$	[121]
*S. cerevisiae*	Cm	$H_{2}$$O_{2}$, MMS, NaAs$O_{2}$	[121]

#### 3.3.4. tRNA Core Modifications in the Response to Antibiotic Stress

Although tRNA modification genes may be directly involved in bacterial antibiotic resistance—e.g., *trmD* introducing $m^{1}$G37 in the anticodon loop (reviewed in [167])—, tRNA core modifications have not been shown to confer resistance to antibiotics. However, a recent pioneering study in *Vibrio cholerae* has uncovered a link between several tRNA modification genes, including those targeting the tRNA core, and the bacterial response to low doses of antibiotics (Table 2) [164]. In this report, initial transposon mutagenesis followed by deep sequencing (TN-seq) [168] in the presence of low-dose antibiotics (sub-minimal inhibitory concentration; sub-MIC) identified several rRNA and tRNA modification genes as important for growth under these antibiotic stress conditions [164]. Several antibiotics were tested, and tRNA modification genes were found to be more largely involved in the response to antibiotics targeting the ribosome, e.g., tobramycin belonging to the aminoglycoside family, than in the response to antibiotics targeting DNA, e.g., ciprofloxacin. These initial genome-wide screens were subsequently complemented with growth competition experiments between the identified tRNA modification deletion strains and the wild-type strain, to determine the effects on the fitness of several antibiotics [164]. Concerning modifications in the tRNA core, under sub-MIC aminoglycoside treatment, deletion of *truB* decreased fitness, while deletion of *dusB* and *trmH* conferred a growth advantage. For other antibiotics, such as ciprofloxacin, trimethoprim and carbenicillin, deletion of *dusB*, *truB*, *trmB* and *trmH* were either decreasing or slightly increasing fitness, depending on the antibiotic considered [164]. These diverse phenotypes for a given gene suggest that the observed effects are not general but antibiotic-specific. Most importantly, this study showed that the effect on fitness in the presence of sub-MIC antibiotics in tRNA modification deletion strains also impacts tolerance to lethal doses of the antibiotics. Although the molecular mechanisms underlying the effects of the queuosine Q34 modification in the antibiotic translational stress response have subsequently been described in detail [169], the mechanisms by which tRNA core modification affects antibiotic tolerance are still elusive, and their study may certainly shed new light on the subtle functions of this group of modifications.

## 4. Conclusions and Perspectives

In this review, we focused on tRNA core modifications, starting from their impact on the structure and dynamics of tRNAs and, subsequently, exploring their reported functions in translation and fitness and their implications in stress adaptation mechanisms. Although the phenotypes associated with particular modifications, or a lack thereof, must ultimately be caused by changes in the structural and/or dynamic properties of the tRNA, thereby altering its ability to interact with cellular partners, what emerges from this review is that the mechanisms by which tRNA core modifications affect fitness, translation and response to stress are not yet fully understood. Although it was not the topic of this review, it is worth mentioning that tRNAs are also implicated in several other stress sensing and response mechanisms, which are not related to post-transcriptional modifications, e.g., the stringent response in bacteria or the nuclear accumulation of tRNAs upon amino acid starvation in yeast [170,171]. In addition, mismethionylation of non-methionine tRNAs confers a protective role against oxidative stress [172,173]. We have also not covered the aspects related to tRNA cleavage upon stress, which is known to be affected by modifications in the ACL region (reviewed in [174]). An important role in stress response mechanisms is therefore occupied by tRNAs, and post-transcriptional modifications are closely involved. This is not surprising given that tRNA modifications are at a crossroads connecting translation with metabolism through their biosynthetic pathways [29]. For instance, the cofactors of tRNA methyltransferases, i.e., S-adenosyl-l-methionine (SAM) and methylenetetrahydrofolate (5,10-methylene-THF), occupy key positions in the one-carbon metabolism, and these metabolites thus affect many aspects of translation, in part through their connections with tRNAs [175]. In recent years, major technological advancements have been made in the fields of mass spectrometry, RNA sequencing, and kinetic studies of translation. These methods have provided, and will continue to provide, important insights into the mechanisms by which tRNA modifications shape gene expression and stress response. In these future studies, although it may be tempting to follow the threads involving anticodon modifications on the pretext that the underlying mechanisms are now better understood, we believe it would be wise not to set aside the threads involving tRNA core modifications.
